# Pyrolysis and gasification at water resource recovery facilities: Status of the industry

**DOI:** 10.1002/wer.10701

**Published:** 2022-03-17

**Authors:** Lloyd J. Winchell, John J. Ross, Dominic A. Brose, Thaís B. Pluth, Xavier Fonoll, John W. Norton, Katherine Y. Bell

**Affiliations:** ^1^ Brown and Caldwell St. Paul Minnesota USA; ^2^ Brown and Caldwell Troy Michigan USA; ^3^ Metropolitan Water Reclamation District of Greater Chicago Cicero Illinois USA; ^4^ Great Lakes Water Authority Detroit Michigan USA; ^5^ Brown and Caldwell Nashville Tennessee USA

**Keywords:** air emissions, biochar, char, energy, energy recovery, gasification, PFAS, pyrolysis, residuals, wastewater

## Abstract

Wastewater treatment generates solids requiring subsequent processing. Costs and contaminant concerns (e.g., per‐ and polyfluoroalkyl substances [PFAS]) are challenging widely used landfilling and land application practices. These circumstances are partly driving the re‐emergence of pyrolysis and gasification technologies along with beneficial reuse prospects of the char solid residual. Previously, technologies experienced operational challenges leading to revised configurations, such as directly coupling a thermal oxidizer to the reactor to destroy tar forming compounds. This paper provides an overview of pyrolysis and gasification technologies, characteristics of the char product, air emission considerations, and potential fate of PFAS and other pollutants through the systems. Results from a survey of viable suppliers illustrate differences in commercially available options. Additional research is required to validate performance over the long‐term operation and confirm contaminant fate, which will help determine whether resurging interest in pyrolysis and gasification warrants widespread adoption.

**Practitioner Points:**

- Pyrolysis and gasification systems are re‐emerging in the wastewater industry.
- Direct coupling of thermal oxidizers and other modifications offered by contemporary systems aim to overcome past failures.
- Process conditions when coupled with a thermal oxidizer will likely destroy most organic contaminants, including PFAS, but requires additional research.
- Three full‐scale facilities recently operated, several in construction or design that will provide operating experience for widespread technology adoption consideration.

## Graphical abstract

Pyrolysis and gasification systems in wastewater treatment applications are garnering renewed interest. Rising costs for solid residual handling, emerging contaminants, and beneficial reuse of residuals provided technology suppliers incentive to overcome historical challenges associated with these thermal processes. This review presents the status of these technologies in the wastewater industry including an in‐depth discussion of system configuration and operating characteristics and fate of emerging contaminants.

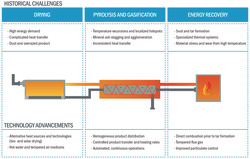

## INTRODUCTION

Wastewater treatment operations generate a solid by‐product requiring further processing before beneficial use or disposal. The wastewater industry often refers to the material collected as sewage sludge; however, after further processing to meet federal and state requirements for beneficial reuse, the sludge becomes classified as biosolids (WEF, 2021).

In 2019, the United States Environmental Protection Agency (USEPA) estimated that 4.75 million dry tonnes of “biosolids” were produced in the United States (US) (USEPA, 2021a) by water resource recovery facilities (WRRF), processing over 3.8 megaliters per day. Roughly 51% of “biosolids” in the US were land applied to recycle the nutrient content and enhance soils. Other practices included reuse or disposal in landfills (22%), incineration (16%), and the remaining 11% using surface disposal sites or other management practices.

Shifts in acceptable land‐use practices, regulations, and public concern for unregulated chemicals have disrupted historical sludge and biosolids applications in the US. For example, increasing population density, regulations, and general aversion to reuse of human waste products have strained the acceptance of sludge or biosolids land application practices (Collins, 2019; Slaughter, 2013). Similarly, a reduction in number (USEPA, 1994) and difficulty in permitting new landfills has led to recent increases in landfill tipping fees in more populated regions of the US (EREF, 2021). In addition, increasing public interest in per‐ and polyfluoroalkyl substances (PFAS) and emerging pollutants has further complicated traditional sludge and biosolids practices (Boxall et al., 2012; Kinney et al., 2006; Navarro et al., 2018; Sepulvado et al., 2011; Walters et al., 2010; Winchell et al., 2022), forcing many municipalities to reconsider end uses.

Pyrolysis and gasification systems are emerging in the wastewater market as thermal treatment processes that could significantly reduce the mass of sludge or biosolids, reducing requirements for off‐site transportation and provide a means for removing or destroying, considered the complete defluorination regardless of carbon oxidation extent, PFAS and other emerging pollutants (Horst et al., 2020; Patel et al., 2020; USEPA, 2021c; Winchell, Ross, et al., 2021). Pyrolysis processes sludge or biosolids in the absence of oxygen, typically at high temperatures (Winchell, Ross, et al., 2021). Gasification is similar but includes substoichiometric oxygen levels and operates at higher temperatures than pyrolysis (Winchell, Ross, et al., 2021). During pyrolysis, sludge or biosolids undergo thermochemical conversion into products representing all three phases—gas, liquid (aqueous or non‐aqueous), and solid (char) (Liu et al., 2017). If controlled streams of a gasifying medium (e.g., air, oxygen, or steam) are introduced into the process, the pyrolysis products will be further refined into a lighter‐molecular weight, non‐condensable off‐gas, also called product gas or syngas (Basu, 2013). The off‐gas can be combusted on‐site or transferred to remote users as an energy source (Basu, 2013; Han et al., 2019) and as a cleaning step prior to releasing the subsequent “flue gas” after the oxidation process to the environment (USEPA, 2021c). Pyrolysis and gasification processing trains show promise for wastewater utilities because PFAS and other emerging pollutants may be removed, and in some cases destroyed, through these high‐temperature processes (USEPA, 2021b; Winchell, Ross, et al., 2021). Still, the efficiency has yet to be documented (Winchell, Ross, et al., 2021). This potential benefit, coupled with the reduction in mass requiring subsequent handling, has driven interest in pyrolysis and gasification as an alternative to historically proven incineration systems (Patel et al., 2020; USEPA, 2021b, 2021c).

This paper provides a current description of pyrolysis and gasification technologies, focusing on US applications. An overview is provided that summarizes the historical challenges for full‐scale implementation and recent advancements in technology deployment. The documented fate of various unregulated chemicals, primarily PFAS, is compiled, and gaps in current understanding are identified through these process trains. This information offers professionals working in the wastewater industry an objective introduction to these technologies for potential applications.

## PYROLYSIS AND GASIFICATION OVERVIEW

Pyrolysis and gasification have long garnered interest for their ability to convert difficult‐to‐handle organic feedstocks into gaseous or liquid fuels that can more easily be stored and used in conventional combustion applications (Bridle & Skrypski‐Mantele, 2004; Haug & Lewis, 2014; Kroll et al., 1983). Additionally, these techniques can process the solid phase material into a carbon‐rich product, called char or biochar, with unique and valuable properties for agricultural and industrial applications (Callegari & Capodaglio, 2018). For example, coal has been gasified since the 18th century, when it was used to produce town gas for street lighting and is still gasified today to produce raw materials for chemical manufacturing (Basu, 2013). Pyrolysis is also used at commercial scale to generate chemical feedstocks, renewable fuel oil, and commercial char soil amendments from various feedstocks, including woody biomass, agricultural residue, and energy crops (Han et al., 2019).

Pyrolysis and gasification have also received strong interest as a thermochemical treatment alternative to incineration (Liu et al., 2020; Safferman et al., 2017; USDOE, 1997; USEPA, 1985). While these technologies can achieve mass reduction comparable to incineration, they require less combustion air and consequently produce less flue gas needing treatment (Winchell et al., In Review). Further, pyrolysis and gasification allow for intermediate treatment, or conditioning, of the off‐gas to remove or recover particulates and acid gases before energy‐producing combustion processes (Asadullah, 2014).

### Thermal reactions

Numerous thermal reactions are involved with pyrolysis, gasification, and combustion, and a high‐level overview of these reactions is presented here. For a more detailed treatment, the reader is directed toward Basu (2013), Boateng (2020), and Higman and van der Burgt (2008) for reviews of the process fundamentals of biomass pyrolysis and gasification.

#### Pyrolysis

When considered as a standalone process, pyrolysis is the thermochemical decomposition of an organic feedstock into a carbon‐rich char and a hydrocarbon‐rich off‐gas. A portion of the off‐gas can be condensed into non‐aqueous (oil or tar) and aqueous phase liquids. Pyrolysis is a prerequisite step to generate the combustible char and off‐gas products from solid or heavy liquid fuels for subsequent oxidation (partial or complete) in gasification or combustion processes (Basu, 2013). The process is conducted in the total, or near‐total, absence of air or oxygen, typically at temperatures between 300°C and 850°C, with the lower end of the range representing the transition from torrefaction (Basu, 2013; Bridle & Pritchard, 2004). The off‐gas contains a diverse mixture of compounds, including hydrogen (H_2_), methane (CH_4_), carbon monoxide (CO), carbon dioxide (CO_2_), acetylene (C_2_H_2_), ethylene (C_2_H_4_), ethane (C_2_H_6_), propane (C_3_H_8_), benzene (C_6_H_6_), and others (Basu, 2013; Liu et al., 2017). The yield of the different products depends on several factors, including feedstock heating rate, catalysts, maximum temperature (or pyrolysis temperature), and residence time and heat distribution in the reactor (Boateng, 2020). The heating rate is often used to distinguish broad categories of pyrolysis (Basu, 2013). Slow pyrolysis is the term used to characterize systems operating at lower heating rates (with corresponding vapor residence times on the order of minutes) which favors char production (Basu, 2013; Boateng, 2020). In fast pyrolysis, residence times are on the order of seconds. In addition to primary feedstock decomposition, secondary decomposition, or “cracking” of the larger molecular weight condensable gases into lighter non‐condensable gas, can occur with extended vapor residence times and higher temperatures (Gao et al., 2014; Han et al., 2019). Secondary cracking can be promoted via the reaction of vapors with heated char, sludge ash, or metallic catalysts (Liu et al., 2022, 2021). Pyrolysis produces a range of products influenced by these environmental conditions including all three material phases—solids, liquids, and gases. Numerous review papers have documented the yield distribution of sludge or biosolids pyrolysis products at various processing parameters with the overall trend being that as pyrolysis temperature increases, the biochar yield (typically between 30% and 50%) decreases with a corresponding increase in gas production (Chen et al., 2014; Gao et al., 2014; Hossain et al., 2011; Jin et al., 2016; Lu et al., 2013; Paz‐Ferreiro et al., 2018; Song et al., 2014; Yuan et al., 2015). The pyrolysis energy reactions will depend on the processing conditions and type of biosolids (Hossain et al., 2009) but often result in a net energy requirement (Daugaard & Brown, 2003; McNamara et al., 2016). As such, the process typically requires supplemental energy for feedstock heating, vaporization of moisture from the feedstock, and reactor radiant heat loss (Ponsa et al., 2017).

#### Gasification

Gasification advances thermochemical transformations beyond those in pyrolysis by reacting char and volatile vapors with a gasifying medium (such as air, oxygen [O_2_], or steam [H_2_O]). Gasification refines gaseous products of pyrolysis into a lower molecular weight fuel. Specifically, the full and partial oxidation of pyrolysis products yields CO and CO_2_, which are then reacted with steam, elemental hydrogen, and carbon (C) in a final reductive (or gasification) zone to generate an off‐gas consisting mainly of CO, H_2_, and CH_4_ (Ahmad et al., 2016; Oladejo et al., 2019). The gasifying medium feed rate and reactor operating temperature control the extent of oxidation, heat release, and limits ash agglomeration and heat production to support endothermic reactions, reactor radiation losses, and latent and sensible heat demands to maintain reactor temperatures at approximately 800–1,000°C (Ahmad et al., 2016; Basu, 2013). A summary of some of the primary reactions within a gasifier is provided (Reactions (1), (4), (5)) (repurposed from Basu, 2013).

(1) $$C+O_{2}\to {\mathrm{CO}}_{2}+\text{heat}\;\;\mathrm{\Delta H}=-394\;\mathrm{kJ}/\mathrm{mol}\;C\;\left(\text{Klass},1998\right)$$

(2) $$C+1/2\;O_{2}\to \mathrm{CO}+\text{heat}\;\;\mathrm{\Delta H}=-111\;\mathrm{kJ}/\mathrm{mol}\;C\;\left(\text{Higman \&}\;\mathrm{van}\;\mathrm{der}\;\text{Bugt},2008\right)$$

(3) $$\mathrm{CO}+H_{2}O\to {\mathrm{CO}}_{2}+H_{2}+\text{heat}\;\;\mathrm{\Delta H}=-41\;\mathrm{kJ}/\mathrm{mol}\;C\;\left(\text{Knoef},2005\right)$$

(4) $$C+{\text{2H}}_{2}\to {\mathrm{CH}}_{4}+\text{heat}\;\mathrm{\Delta H}=-75\;\mathrm{kJ}/\mathrm{mol}\;C\;\left(\text{Klass},1998\right)$$

(5) $$\begin{array}{rcll} C+{\mathrm{CO}}_{2}+\text{heat} & \to & \text{2CO}\;\mathrm{\Delta H}= & +172\;\mathrm{kJ}/\mathrm{mol}\;C\left(\text{Higman \&}\;\mathrm{van}\;\mathrm{der}\;\text{Bugt,}\;2008\right) \end{array}$$

### Contemporary systems

Contemporary pyrolysis and gasification systems treating sludge or biosolids must address operational issues associated with systems from the past. Today, active installations have addressed historical shortcomings by simplifying the process, repurposing demonstrated technology components, and improving system controls per the following discussion.

#### Historical lessons

Sludge and biosolids pyrolysis and gasification systems in operation or under development today in the US represent the evolution of the technology from its initial application in the early 1970s. To the authors' knowledge, the first commercial‐scale use of pyrolysis or gasification with sludge was deployed at the Central Contra Costa Sanitary District (Brown and Caldwell, 1976) when the WRRF's two multiple hearth furnaces were run in oxygen‐deficient conditions during facility startup in 1975. Later, two 36‐dry tonnes per day (dtpd) multiple hearth furnaces were installed at the Arlington County, Virginia Advanced Water Treatment (AWT) Facility in 1983 capable of incinerating or gasifying sludge by limiting combustion air input and oxidizing the off‐gas in an afterburner with waste heat recovery (Kroll et al., 1983). Full‐scale operation of these furnaces demonstrated that gasification of sludge was possible. However, both WRRFS ultimately operated the systems in incineration mode due to the additional complexity and cost of maintaining the afterburner and heat recovery systems.

In a similar timeframe, the city of Los Angeles constructed an extensive sludge gasification system with three trains, each with a capacity of 120 dtpd. The system was noted to have operated for 10 years until being decommissioned due to difficulties with the Carver‐Greenfield drying process and other considerations (Haug & Lewis, 2014).

Several lessons learned at a pyrolysis plant in Australia were identified (Bridle & Skrypski‐Mantele, 2004). First, the upstream solids processing performance must be verified as the sludge quality variability required a capital upgrade of the dryer process. Operators of the pyrolysis system benefit from an industrial background to reliably manage the system, including all related processes. Tubes in the off‐gas condensing equipment fouled and were replaced with a direct spray system. Regardless, the downstream oil–water separator still required monthly cleaning. The authors noted other issues with char handling and emergency venting that required ancillary equipment modifications and replacement.

A more recent gasification example includes the 160 wet tonnes per day (wtpd) system at the city of Sanford, Florida; the facility, owned by Maxwest Environmental Systems, Inc., was operated from 2009 to 2014 (Snyder, 2015). While initial operational issues required costly modifications that ultimately led to the facility's closure, the modifications did result in a technology configuration that achieved stable operations, albeit for a limited time. Specifically, the dryer and dried product delivery system were changed from batch to continuous feed to stabilize process loading and off‐gas production. The gasifier reactor was also changed from a fixed bed updraft configuration to a fluidized bed to improve heat transfer and temperature control. Discussion with a technology provider familiar with the facility's operation also identified system shutdown as an additional challenge (McGolden, 2021). The unit had to be shut down with product retained inside to protect the uninsulated steel floor from exposure to high temperatures. This resulted in air intrusion and “burn out” of the product, reaching high temperatures that would melt the resulting ash into slag that required chipping out before starting up again.

KORE Infrastructure completed a 6‐year demonstration test of biosolids pyrolysis in 2014 at the Los Angeles County Sanitation District's Joint Water Pollution Control Plant in Carson, California. The project's primary finding was that system maintenance and rehabilitation requirements during operation were primarily associated with hydrogen sulfide corrosion from the pyrolysis off‐gas (Wirtel, 2021). High levels of hydrogen sulfide were observed in the pyrolysis off‐gas and KORE Infrastructure noted that their plans for future, permanent installations will include a detailed pyrolysis off‐gas characterization and materials selection assessment to address hydrogen sulfide corrosion potential.

Logan City Council (2021) in Queensland, Australia, recently conducted a biosolids gasification demonstration project at the Loganholme Wastewater Treatment Plant between January and August 2020. During the project, 12 test runs of a multiple hearth gasifier manufactured by Pyrocal Pty Ltd. were operated at a dried‐biosolids feed rate of 480 kg/h (74% of maximum capacity at 650 kg/h). Over the longer duration runs (100 h), system throughput was reduced due to soot and tar build‐up in the air manifolds. However, the soot and tar readily burned off when the feed was paused, and an automated burn‐off sequence is planned for future operation at full scale. Additionally, the pyrolysis off‐gas' non‐sticky carbon and dust carryover overwhelmed the original spray absorber scrubber and barrier filter located after the directly coupled thermal oxidizer. As a result, the system was modified to include a Venturi device at the front of the scrubber instead of the barrier filter. In addition, a wet electrostatic precipitator (WESP) is planned for inclusion at full scale for improved dust control.

Newer generation pyrolysis and gasification systems have incorporated these lessons learned into their design and operation by improving construction materials, simplifying design of energy recovery systems (i.e., using air and hot water mediums in lieu of thermal oil or steam), and integrating demonstrated technology components (i.e., dust control and product feeding subsystems) with modern instrumentation and controls to improve reliability (McGolden, 2021; Mooney, 2021; Villa, 2021). While substantial progress has been made in these systems, further evidence of successful operation over the long term is required before they can be considered proven at commercial scale.

#### Active installations

As discussed previously, application of sewage sludge or biosolids pyrolysis and gasification has been limited so far in the US. At the time of writing, the authors identified three commercial‐scale facilities in operation processing sludge in the US (Table 1).

**TABLE 1:** Commercial pyrolysis and gasification facilities currently processing sludge in the United States

Location	Technology vendor	Commissioned	Rated capacity	Maximum energy production	Reported mass output
Morrisville Municipal Authority, Pennsylvania^a^	Ecoremedy Fluid Lift Gasification™ (Ecoremedy, 2021)	2019	32 wtpd (27% total solids)	2640 MJ/h heat in process air for thermal drying	2.4 wtpd
Silicon Valley Clean Water Authority, California	BioForceTech Corporation BioDryer and Pyrolysis (BioForceTech Corporation, 2022)	2018	14 wtpd of dewatered, digested biosolids (20% total solids)	320 MJ/h heat in hot water for drying	1.1 wtpd
City of Lebanon, Tennessee	Aries Clean Technologies Downdraft Gasification (Rulseh, 2018)	2016	29 wtpd of blended waste wood, scrap tires, and dewatered, digested biosolids	420 kW of electricity from flue gas driven organic Rankine cycle generator	1.5 wtpd

^a^ System demonstration recently completed, and equipment decommissioned.

**FIGURE 1:**
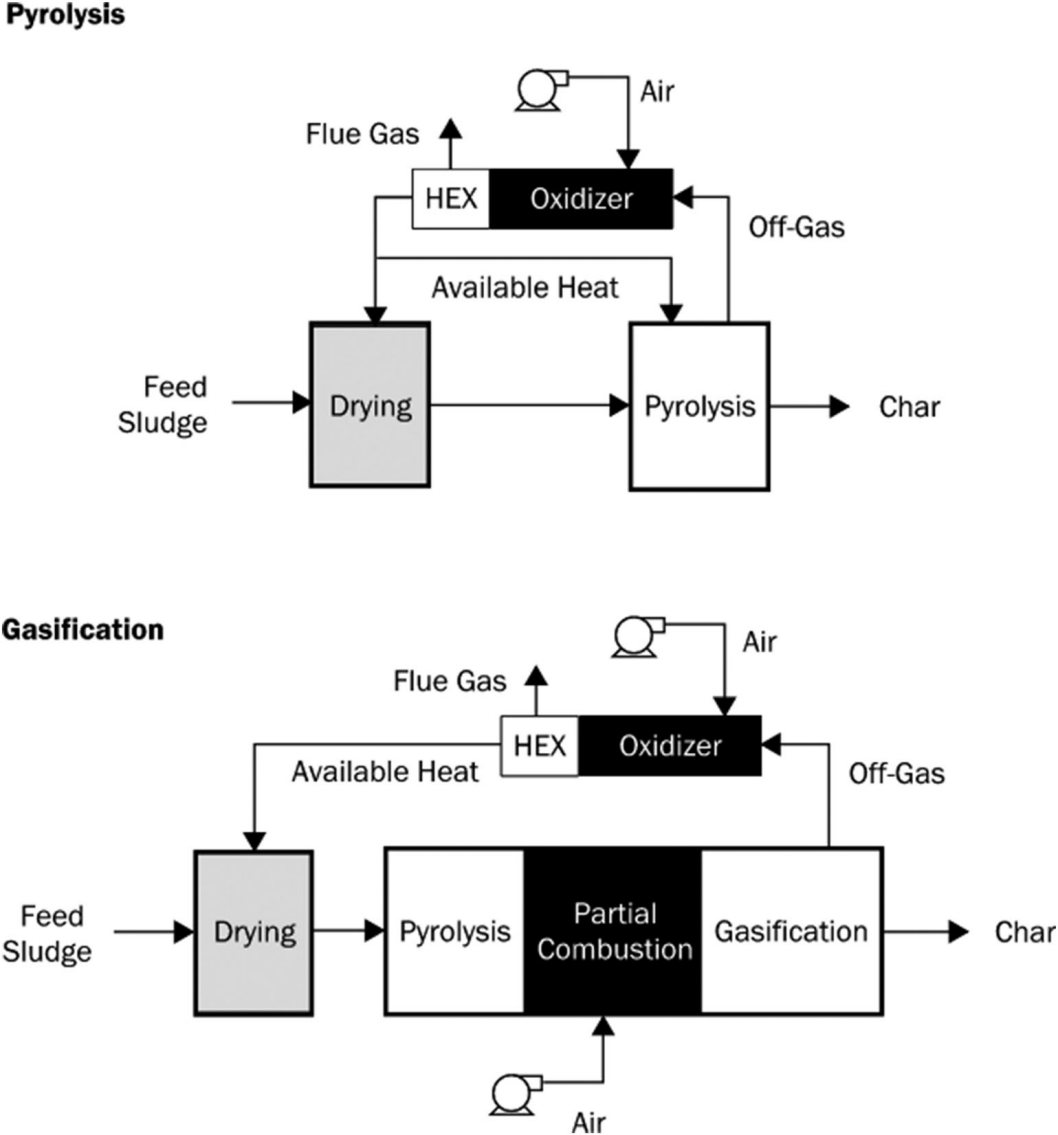
Pyrolysis and gasification process schematics illustrating the various sub‐processes and differentiating use of a gasifying medium, air for this example

All the systems noted in Table 1 consist of three core unit processes, schematically represented in Figure 1. The first process dries the sludge or biosolids to the desired moisture content. The system then processes the dried product through the thermal reactor where pyrolysis and, if intended, gasification occur. Finally, the off‐gas from the thermal reactor is combusted in a thermal oxidizer for energy recovery and air emissions control. Sub‐unit processes include product feeding, residual char handling from the thermal reactor, and energy recovery systems. The following discussion and later technology comparison section discuss these processes in more detail.

#### Moisture reduction

The existing facilities demonstrate two approaches to reducing moisture—a critical preparation step in sludge or biosolids pyrolysis or gasification. The Ecoremedy and Bioforcetech facilities use a discreet upfront drying step. The Ecoremedy technology uses a single‐pass rotary drum dryer to produce a dried pellet meeting Class A requirements under the USEPA biosolids regulations 40 C.F.R. § 503 (Ecoremedy, 2021; USEPA, 1993). The Bioforcetech pyrolysis facility uses batch‐fed biodryers to reduce moisture content through heating, applied via an initial, exothermic composting step, and subsequently from an auxiliary hot water system (BioForceTech Corporation, 2022). The Aries Clean Technologies gasification facility takes a different approach by using wood waste and scrap tire feedstocks as bulking agents to reduce the moisture content of the blended feed, which allows for the recovery of additional energy from the gasification of the bulking agents (Rulseh, 2018). Traditionally, thermal drying can be a costly, complex, and energy‐intensive process (WEF, 2018), and the use of bulking agents represents an opportunity to eliminate this step. However, feedstock blending with a downdraft gasifier limits biosolids content to approximately 10% of the blended feedstock mass, which requires ongoing coordination with third‐party suppliers (Rulseh, 2018).

Each of these facilities processes a sludge that has undergone aerobic or anaerobic biological treatment before drying. Minimizing or eliminating upstream stabilization presents an opportunity to increase the energy density of the feed sludge, especially for feedstocks containing primary sludge from wastewater treatment. However, stabilization provides equalization in flow and loading to thermal drying processes. In addition, it safeguards the system from high levels of odors, internal product adhesion, and reactor fouling, which have been associated with dryer operations with unprocessed primary sludge (WEF, 2018). Consequently, advancements in drying technology and system design will be required to reliably apply pyrolysis and gasification to unstabilized sludge.

#### Thermal reactor

A variety of thermal reactor types exist for pyrolysis and gasification. A brief description of reactor types applied to wastewater sludge and biosolids at a commercial scale is provided here. The reader is referred to Gao et al. (2020), Patel et al. (2020), and Basu (2013) for a more comprehensive discussion of reactor types and processing parameters. Landen (2018) completed a survey of approximately 200 biomass pyrolysis and gasification manufacturers worldwide and identified four primary reactor types used by manufacturers operating at least five systems at commercial scale (10,000 tonnes capacity per year) globally. A summary of the findings, updated with more recent technology installations, is provided here.

Landen (2018) identified three reactor concepts for biomass pyrolysis relevant to sludge or biosolids: a heated reactor with an auger, a reactor with a heated auger, and a rotary kiln. The study also identified multiple reference installations of batch kiln reactors used to produce charcoal from wood biomass; however, the batch process included a high operational and footprint demand that can be prohibitive for WRRFs.

The heated reactor with auger, as manufactured by PYREG GmbH and packaged by BioForceTech Corporation, uses a double‐wall reactor where hot exhaust from direct combustion of the pyrolysis off‐gas is passed through the outer chamber to heat the wall of the inner chamber (BioForceTech Corporation, 2021). The feedstock is transferred along the inner chamber via a shafted screw auger and absorbs heat from the wall while undergoing pyrolysis. KORE Infrastructure manufacturers a pyrolysis system with a similar concept, where two horizontal augured reactors are operated in series inside a chamber heated externally with hot flue gas from the pyrolysis off‐gas burner (Kore Infrastructure, 2021). Feedstock characteristics are required to be kept within a specific operating range to absorb heat evenly along reactor walls to guard against material stress and ash agglomeration from localized hot spots.

ETIA Ecotechnologies alternatively uses an electrically heated, shaftless screw auger to convey and heat biomass along an insulated reactor, improving the system's capacity for temperature control but eliminating the ability to meet process heat demands by direct combustion of the pyrolysis off‐gas (ETIA Ecotechnologies, 2021).

The third reactor type transfers biomass along an externally rotated kiln with an external heating chamber. CHAR Technologies Ltd. is one representative manufacturer of this rotary kiln pyrolysis system that uses multiple pyrolysis off‐gas burners to provide process heat at controlled rates to the heating chamber and requires off‐gas cleaning prior to combustion in the burner system, creating the potential for tar condensation and associated reduction in heating value of the off‐gas (CHAR Technologies, 2021). However, the rotary kiln eliminates the need for moving parts in the hot zone of the reactor and the associated concern for material stress and wear.

Green Waste Energy employs a different technology using a tower reactor that introduces the feedstock at the top, which falls and undergoes pyrolysis treatment (Green Waste Energy, 2021). Treated off‐gas recycles to burners installed on the tower jacket to satisfy heat requirements. Landen (2018) established that a critical feature of all pyrolysis reactor types is their ability to minimize or eliminate contact between pyrolysis off‐gases and biochar to preserve characteristics of the latter.

In contrast to the indirectly heated pyrolysis reactor systems described above, gasification derives its process heat from the limited combustion reactions conducted within the reactor. Consequently, gasification systems are less dependent on reactor surface area, a feature that Landen (2018) identified as a limiting factor for scaling up pyrolysis reactors.

The introductory location of the combustion air, or gasifying medium, is critical for heat distribution within the reactor and is a primary differentiator between gasifier reactor configurations (Basu, 2013). The feedstock and gasifying medium enter the reactor from opposite ends in updraft gasifiers. The pyrolysis process is allowed to advance to separate off‐gas from char, which then contacts with combustion air. As the oxygen is depleted, the gas moves toward the feedstock entrance, transferring sensible heat and undergoing reductive, gasification reactions.

Landen (2018) identified the moving grate gasifier, manufactured by Ecoremedy LLC, as a promising reactor type, in which combustion air is injected beneath a grate used to advance biomass through the process. The air is injected in several zones, each of which can be modeled as individual updraft gasifiers to control system temperature and char oxidation.

The gasifier manufactured by Pyrocal Pty Ltd. uses a multiple hearth reactor that transfers biomass by rotating rake arms and introduces combustion air in the final zone where the hot gases pass up through the reactor (Logan City Council, 2021). In downdraft gasifiers, the feedstock and oxidizing medium are introduced at the reactor entrance to promote immediate contact with pyrolysis off‐gas, promoting pyrolysis off‐gas cracking and tar reduction.

A similar concept is employed in fluidized bed reactors where the air and feedstock are reacted together in an inert fluidized bed to promote mixing and heat transfer. The design subjects the gas stream to gasification reactions as it travels toward reactor zones where the oxygen has been consumed. The fluidized bed reactor allows for operation at larger scale, as evidenced by the current development of two large fluidized bed gasification facilities by Aries Clean Technology in urban centers in the US (Aries Clean Technologies, 2021).

#### Energy recovery

Energy recovery is currently employed in pyrolysis and gasification facilities that process waste streams. The energy recovery step is typically achieved through onsite, direct‐combustion of the off‐gas instead of processing it for use off‐site chemical or fuel production. Product conditioning to produce an off‐site chemical or fuel (most commonly a bio‐oil from pyrolysis and syngas from gasification) is a highly complex field of study. Specialized knowledge is required to design and operate these systems (Han et al., 2019).

One concern of pyrolysis and gasification is the resulting condensable fraction (aqueous or non‐aqueous) of the off‐gas, commonly referred to as tar, that forms as a liquid in low‐temperature zones of a reactor and downstream gas handling equipment (Ponsa et al., 2017). Tar is a viscous liquid that can plug downstream passages and energy conversion equipment, including gas engines (Basu, 2013). Tar production can be minimized through reactor design and off‐gas cleaning; however, off‐gas cleaning processes impart an operational and parasitic energy demand which can result in difficult to treat waste streams (Basu, 2013).

Alternatively, tar production can be induced under controlled conditions for energy recovery (Gao et al., 2020; Haghighat et al., 2020; Hossain et al., 2009; Kim & Parker, 2008). However, handling of the liquid material introduces unique challenges. The higher oxygen content in biomass feedstocks compared to fossil fuels results in a bio‐oil product that is less energy‐dense, potentially unstable in combustion applications, corrosive, and increasingly viscous over time (Basu, 2013).

Thermal oxidizers (direct combustion) employed at the three existing, commercial‐scale facilities combust raw off‐gas from pyrolysis or gasification directly before tar has a chance to form (BioForceTech Corporation, 2022; Ecoremedy, 2021; Rulseh, 2018). Thermal oxidizers mitigate operation risks and convert all the chemical energy stored in the condensable and non‐condensable fractions of the off‐gas (Niessen, 2002). The heat from thermal oxidation can be transferred through heat exchangers to process air, hot water, steam, or drive an organic Rankine cycle (ORC) generator, which is used at the Aries Clean Technologies facility. A schematic of the sequential processes occurring with pyrolysis and gasification followed by direct combustion is provided in Figure 1.

The defining question for the energy balance of each system is whether the available heat recovered from combustion is sufficient to meet the demands for upfront drying and internal heat sinks. As conventional thermal drying has historically required a large amount of fuel, drying with pyrolysis and gasification requires a relatively high level of conversion efficiency to achieve autothermal operation (WEF, 2018).

**FIGURE 2:**
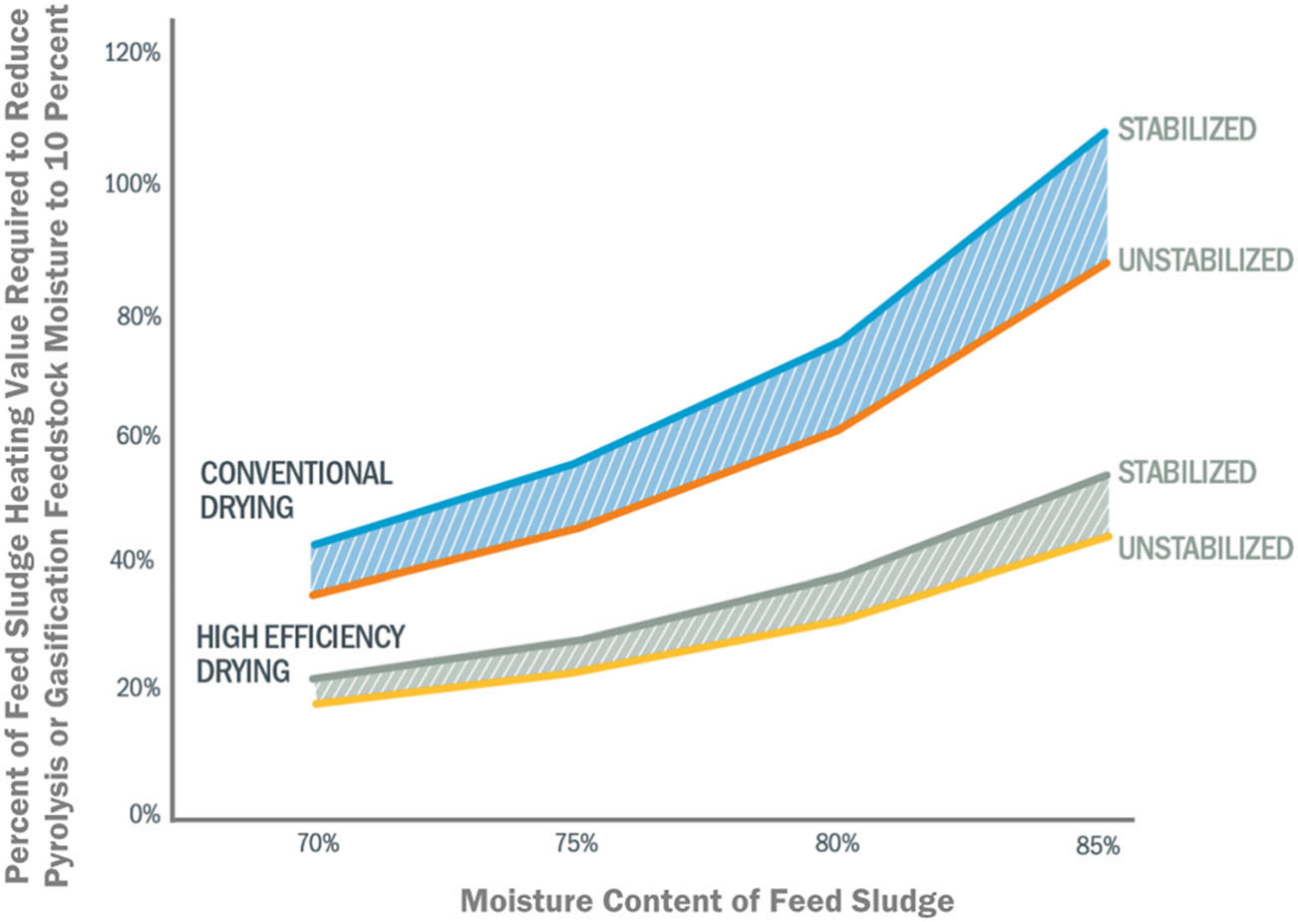
Net percent heat recovery from feed sludge chemical energy required to reduce pyrolysis or gasification feedstock moisture to 10%. Assumes (a) thermal efficiencies for conventional and developing high‐efficiency dryers of 3.0 and 1.5 kJ‐heat/g‐water, respectively (BioForceTech Corporation, 2022; WEF, 2018); (b) input sludge higher heating value of 23.8 kJ/g‐volatile solid (Niessen, 2002); and (c) volatile solid contents of 65% and 80% for stabilized and unstabilized sludge, respectively (WEF, 2018)

A conceptual relationship between the efficiencies required for converting chemical energy in sludge or biosolids to meet the heat demands of conventional and developing high‐efficiency dryer technologies (e.g., biodryers) is presented in Figure 2. While unreacted carbon in char limits recoverable heat, as well as various other heat sinks, direct combustion of pyrolysis and gasification off‐gases, theoretically, can exceed the heat needed for drying; however, the net impact has not yet been quantified, as operational data from full‐scale facilities are not yet publicly available. Future work is required to assess operational data to measure and verify critical process variables and energy performance indicators, such as percent available heat recovery from feedstock, to verify successful operation.

#### Operating conditions

Given that these technologies are emerging in the wastewater marketplace, several equipment suppliers were surveyed to document operating conditions. Table 2 summarizes the information collected based on the dewatered solids characteristics footnoted. The unit feed rate ranged broadly with capacities capable of handling solids output from most WRRFs with a single train.

**TABLE 2:** Thermal treatment operating conditions^a^

Parameter	Units	Supplier 1	Supplier 2	Supplier 3	Supplier 4	Supplier 5
Process	–	Gasification	Pyrolysis	Pyrolysis	Gasification	Pyrolysis
Unit feed rate range	Dry tonne/d	6.1–24.4	2.1–6.8	22–110	22–90	6.7–56.7
Dryer						
Type	–	Rotary drum	Rotary cylinder	Belt in tandem with rotary drum	Rotary drum	Rotary drum
Target total solids	Percent	92	80	90	90	60
Temperature	°C	535 inlet 100 outlet	65	80–105	510 inlet 87 outlet	800 inlet 110 outlet
Solids residence time	min	15	3,330	220	20	20
Evaporative capacity	kg H_2_O/h	2,720	720	14,500	13,600	4,960
Thermal efficiency	kJ/kg of H_2_O	3,400	1,939	N/P	2,775	2,685
Supplementary fuel	kJ/h per dry tonne/d	0	0	0	0	45,100
Recycled energy input^b^ ^,^ ^c^	kJ/h per dry tonne/d	281,100	296,000	N/P	416,200	168,000
Reactor						
Type	–	Moving chain grate	Inclined screw	Passive falling tower	Fluidized bed	Rotary kiln
Temperature	°C	750	620	950	680	650–850
Gas residence time	s	1	7–8.5	10	8–10	1.2
Solids residence time	min	90	15	15	20	20
Stoichiometric air	–	0.3	0.0	0.0	0.32	0.0
Supplementary fuel	kJ/h	0	0	0	0	0
Recycled energy input^b^ ^,^ ^c^	kJ/h per dry tonne/d	0	76,300	N/P	0	190,400
Thermal oxidizer						
Type	–	Proprietary	Flameless direct fired	Regenerative thermal oxidizer	Direct fired	Staged air cyclone
Temperature	°C	1,200	980	850	980	850
Gas residence time	s	2	2.5–3.5	2.5	1–2	2
Flue gas flow rate^b^	Nm^3^/h per dry tonne/d	300	180	N/P	400	330
Supplementary fuel^b^ ^,^ ^d^	kJ/h per dry tonne/d	0	0	0	0	2,400
Stoichiometric air	–	1.15	1.15	N/P	2.9	N/P
Energy recovered^e^	Percent of available from flue gas sensible heat	79	71	70 ^f^	75	65 ^g^
Major motor requirements^b^	kW/dry tonne/d	4.7	23.5	1.8	15.7	4.0
Solid residual						
Production	Percent of dry feed	25	45	N/P	27	35.8
Combustible fraction	Percent	0	10	N/P	14	16–30
Carbon content	Percent	0	30	<2	14	15–25
Nitrogen content	Percent	0	3	minimal	minimal	0

Abbreviations: N/A, not applicable; N/P = not provided.

^a^ Values based on the following dewatered solids characteristics: total solids—28%; combustible solids (CS)—75%; higher heating value—23,260 kJ/dry kg CS; carbon—57% of CS; hydrogen—7% of CS; oxygen—30% of CS; nitrogen—5% of CS; sulfur—1% of CS.

^b^ Normalized to feed rate at maximum size offered by the equipment supplier.

^c^ Heat recycled to sustain the process.

^d^ Natural gas equivalent.

^e^ Amount required to self‐sustain process.

^f^ Author‐calculated value based on 5 MW power generation quoted by the supplier at 204 dry tonne/d at 20% total solids and assuming 40% power production efficiency, combustible solids, and heating values as noted. Value is conservative as it ignores energy radiation losses, latent heat of vaporization for water resulting from the combustion of off‐gas, and heat demand to raise combustion air to process temperature.

^g^ Supplier recycles a portion of energy as cleaned off‐gas in addition to heat recovery from the flue gas. Value estimated using the heating value of cleaned off‐gas, actual energy recovered percentage is higher if the latent heat of water vapor from combustion is included but was not available.

Dryer operation varied the most between vendors compared to the other unit processes. Solids residence time in the dryers reflected the operating temperatures (i.e., increasing time with lower temperature). Supplier 2 differed the most from others based on the biologically driven heating concept resulting in the highest energy efficiency but the longest solids residence time. Overall, dryers required the bulk of the energy recycled in the process.

Thermal reactor operation values were more consistent across the suppliers surveyed. Target operating temperatures ranged over 300°C, but vendors noted the value must be modified depending on the off‐gas and char production goals. Gas residence times also appeared supplier‐tailored and not technology‐dependent as some operated at approximately 1 s while others approached 10 s. Suppliers reported similar solids residence times except Supplier 1, which processed up to six times longer. Pyrolysis systems did require recycled energy to maintain operating temperatures where the gasifiers used the heat released from internal partial combustion.

Each supplier provided the thermal oxidizer to destroy pollutants and liberate heat from the hydrocarbon‐rich off‐gas combustion. The suppliers operated at similar temperatures and residence times, except Supplier 1 exceeded the temperature range provided by the others. This high temperature is conceivable given the relatively low stoichiometric air requirement, which acts as a heat sink.

The resulting char characteristics in Table 2 are an example only. Each supplier can change their process to shift the char to either more or less carbon‐rich depending on the site‐specific goals. For example, Supplier 1 typically converts the feedstock to ash compared to Supplier 2 who intentionally produces a char product with high carbon content. A higher carbon char reduces the amount of heat released during the thermal conversion and can lead to supplemental fuel requirements but provides a potential means for long‐term carbon sequestration in the char product (Callegari & Capodaglio, 2018; Patel et al., 2020; Racek et al., 2020). Alternatively, a lower carbon char reduces the mass of residual product needing to be managed.

#### Char

During pyrolysis and gasification, off‐gas production increases as temperatures increase, while char yields decrease (Song et al., 2014; Yuan et al., 2015). Sludge‐ and biosolids‐derived char have been widely studied for their beneficial reuse potential, primarily as soil amendments for turfgrass and agricultural crop applications (Callegari & Capodaglio, 2018). Char is a beneficial soil amendment; however, properties important to soil quality, such as pH, cation exchange capacity, and nutrients, can vary widely and are dependent on feedstock and pyrolysis temperatures (Al‐Wabel et al., 2018). Additionally, while char can be described as a material derived from sludge or biosolids, as defined in USEPA's biosolids regulations 40 C.F.R. §503 (USEPA, 1993), it may be a lengthy process for producers to receive recognition of char as an Exceptional Quality or Class A biosolids product from regulators and use it as a soil amendment.

Char from a range of feedstocks, including sludge and biosolids, has been shown to have a liming effect on soils, improve water holding capacity, and increase crop nutrient availability (Jeffery et al., 2011; Jellali et al., 2021; Racek et al., 2020). Increasing pyrolysis temperatures increase sludge‐ and biosolids‐derived char pH, surface area, pore‐volume, total phosphorus (TP), and potassium (K) concentrations and decrease nitrogen (N) concentrations (Table 3). Therefore, targeting specific char characteristics could be achieved by using select temperatures. For example, land applying biosolids‐derived char (produced at 300°C and 500°C) at 15‐tonnes/ha to a moderately acidic soil increased pH, TP, and corn grain yield and did not result in an accumulation of heavy metals, indicating that char could be a suitable replacement for mineral fertilizers (de Figueiredo et al., 2019, 2020). Sludge‐derived char (produced at 600°C) applied at 1.7‐tonnes/ha was also beneficial to moderately alkaline soils, increasing TP and K concentrations and corn yield while decreasing the uptake of metals (Xie et al., 2021).

**TABLE 3:** Chemical properties of sludge‐ and biosolids‐derived char

Parameter^a^	Unit	Biosolids/sludge	300°C	400°C	500°C	600°C	700°C	Land Application Limits EQ/Ceiling
pH^b^ ^–^ ^j^		4.4–7.2	5.3–7.3	4.9–8.5	6.5–9.8	8.1–12	8.4–12	
Surface Area^b^ ^,^ ^e^ ^–^ ^j^	m^2^/g	2.2–18	5.3–20	0.1–23	3.2–52	12–27	27	
Carbon^b^ ^–^ ^f^ ^,^ ^h^ ^,^ ^j^	wt.%	21–38	23–27	20–23	18–21	20–21	20	
Nitrogen^b^ ^–^ ^f^ ^,^ ^h^ ^–^ ^j^	wt.%	3.0–5.4	3.3–6.1	2.4–3.8	1.8–3.1	1.5–2.7	0.91–1.2	
Phosphorus^b^ ^,^ ^e^ ^,^ ^f^ ^,^ ^h^ ^–^ ^j^	wt.%	1.5–5.2	3.5–4.1	3.4–4.3	3.6–6.1	4.5	4.9	
Potassium^b^ ^,^ ^e^ ^,^ ^f^ ^,^ ^h^ ^–^ ^j^	wt.%	0.08–0.75	0.11–0.75	0.22–0.90	0.13–1.0	0.26–1.3	1.7	
Sulfur^b^ ^–^ ^f^ ^,^ ^j^	wt.%	0.67–5.2	0.62–4.5	0.61–4.7	0.50–5.9	0.55–0.87	6.2	
Zinc^b^ ^–^ ^j^	mg/kg	306–2,580	321–1,417	986–2,572	411–2,822	1,090–3,368	1,090–2,175	2,800/7,500
Copper^b^ ^–^ ^j^	mg/kg	115–1,218	152–1,150	213–1,551	138–1,674	209–1,697	227–1,500	1,500/4,300
Lead^b^ ^–^ ^j^	mg/kg	20–3,740	115–4,410	83–4,900	93–5,120	111–5,250	132–5,200	300/840
Nickel^c^ ^–^ ^e^ ^,^ ^g^ ^–^ ^j^	mg/kg	23–112	50–182	95–165	35–292	101–219	103–195	420/420
Cadmium^c^ ^,^ ^e^ ^–^ ^j^	mg/kg	BDL − 169	2.6–197	2.8–225	3.2–235	229	3.2–123	39/85
Arsenic^c^ ^,^ ^e^ ^,^ ^h^ ^,^ ^i^	mg/kg	<3–26	<3–27	<3–31	<3–32	35	<3–37	41/75
Selenium^c^	mg/kg	<6.6	<6.6	<6.6	<6.6	<6.6	<6.6	
Chromium^b^ ^–^ ^e^ ^,^ ^h^ ^–^ ^j^	mg/kg	20–449	79–108	61–665	61–1,065	106–1,374	83–103	
Manganese^b^ ^,^ ^d^ ^,^ ^f^ ^,^ ^j^	mg/kg	56–748	58–494	536–731	80–1,076	1,383		
Cobalt^b^ ^,^ ^e^ ^,^ ^j^	mg/kg	2.1–20	16–22		19–25			
Reference(s)		^b^ ^–^ ^i^	^b^ ^,^ ^c^ ^,^ ^f^ ^,^ ^i^ ^,^ ^j^	^c^ ^,^ ^d^ ^,^ ^f^ ^,^ ^h^ ^,^ ^i^	^b^ ^–^ ^d^ ^,^ ^f^ ^–^ ^j^	^d^ ^,^ ^f^ ^,^ ^i^	^c^ ^,^ ^i^	USEPA (1993)

^a^ Showing range of reported values for biosolids/sludge and different pyrolysis temperatures. BDL = below detention limit. EQ = exceptional quality.

^b^ de Figueiredo et al. (2019).

^c^ Hossain et al. (2011).

^d^ Jin et al. (2016).

^e^ Khan, Chao, et al. (2013).

^f^ Lu et al. (2013).

^g^ Méndez et al. (2012).

^h^ Song et al. (2014).

^i^ Yuan et al. (2015).

^j^ Chagas et al. (2021).

In char, nutrients, such as N, phosphorus (P), K, and sulfur (S), follow different fate pathways during thermal processing. Up to 40% of the N in biosolids can be lost to the gas phase, primarily as ammonia and hydrogen cyanide, at temperatures up to 800°C (Chen et al., 2011; Wei et al., 2015). Similarly, Hossain et al. (2011) found that up to 40% of S volatilized with increasing temperatures. P and K, however, become concentrated in char on a mass concentration basis as biomass is lost (Table 3; Lu et al., 2013; Yuan et al., 2015). TP concentrations in biosolids‐derived char increased by 40% to 50% at 700°C, indicating P was associated with the inorganic fraction of biosolids (Hossain et al., 2011; Yuan et al., 2015).

The pyrolysis and gasification of sludge and biosolids volatilize a small portion of heavy metals in the feedstock; however, the remainder is concentrated in the char due to the loss of biomass (Chanaka Udayanga et al., 2018). Pyrolysis of sludge and biosolids results in a reduction of leaching and bioavailability to plants relative to the feedstock (Jin et al., 2016; Lu et al., 2016). Méndez et al. (2012) demonstrated that sludge pyrolysis decreased the plant‐available and mobile forms of nickel (Ni), zinc (Zn), copper (Cu), and lead (Pb). When blended with agricultural soil, the leaching of Cu, Ni, cadmium (Cd), and Zn from char was lower relative to raw sludge. This enhanced sorption is attributed to the large surface area, porous structure, and complexation with surface functional groups and has also been shown to reduce the uptake of polycyclic aromatic hydrocarbons (PAHs) by plants and remove micropollutants, including metals, hormones, and pharmaceuticals and personal care products (PPCPs) from wastewater (Khan, Wang, et al., 2013; Kimbell et al., 2018; Tan et al., 2015; Tong et al., 2019).

Char has agronomic value, mainly due to the carbon, nutrients, and liming effect when blended with soils. Char has also been shown to have carbon capture and sequestration benefits that are being investigated (Callegari & Capodaglio, 2018; Patel et al., 2020). Char may also be used directly in wastewater treatment, taking advantage of its contaminant sorption properties (Xie et al., 2021); however, Tong et al. (2016) found that the sorption capacity of biochar for triclosan in secondary effluent was suppressed due to the presence of total suspended solids and other organic constituents. Racek et al. (2020) noted that char from organic material is too valuable for land application and can be used to store volatile nutrients, as an absorber for removing odor, insulating material in the building industry, energy storage in batteries, and filters for landfills. Because of the potential value of char, several pyrolysis system suppliers indicated that they would manage char at no cost, and one indicated the potential for profit‐sharing.

#### Air emissions

Pyrolysis and gasification technologies include a gas‐phase output that requires treatment to meet air emissions regulations. WEF (2009) provided a general overview of the permitting process for sewage sludge incinerators which would generally apply to pyrolysis and gasification systems. Pyrolysis and gasification technologies are not classified under the USEPA (2011) Sewage Sludge Incineration Rule, 40 C.F.R. § 60, but each installation requires a site‐specific applicability determination ruling from the USEPA. However, recent action by the USEPA may lead to the promulgation of future regulations (USEPA, 2021b) for these technologies.

Contemporary air pollution control systems can be configured to meet regulatory emissions limits. System suppliers have a wide array of air pollution control equipment options to meet emission criteria. Potential equipment for use with pyrolysis and gasification systems is covered in depth in combustion‐based references (Niessen, 2002; WEF, 2009). The pyrolysis facility currently operating in the US and the gasification facility that recently completed its trial operation both employ similar air pollution control equipment to meet regulatory limits and prove system performance for future applications. In either case, the thermal oxidizer combusts the off‐gas from the thermal reactors, converting organic pollutants to CO_2_ and H_2_O at high efficiency (Niessen, 2002). Subsequently, wet scrubbers collect particulate and acid gases. The gasification facility uses a cyclone upstream of the wet scrubber to capture particulates. Before atmospheric discharge, an activated carbon filter provides the final cleaning step—mercury (Hg) and trace organic compound removal.

By operating at substoichiometric oxygen levels, pyrolysis and gasification technologies offer a unique opportunity to minimize nitrogen oxide (NO_X_) emissions. In contrast, other common pollutants require air pollution control processes to meet regulatory limits (Winchell et al., In Review). Thermal‐ and fuel‐bound mechanisms produce NO_X_ in thermal processes, with the former being insignificant at temperatures less than 1,093°C when processing WRRF sludge or biosolids (WEF, 2009). The fuel‐bound mechanism requires oxygen in the presence of N in the sludge or biosolids to produce NO_X_. Fuel‐bound N primarily converts to N_2_ and ammonia (Basu, 2013). Pyrolysis and gasification systems may also sequester some N in the char (Tables 2 and 3), limiting NO_X_ emissions.

## UNREGULATED CHEMICAL REMOVAL AND DESTRUCTION

The potential for pyrolysis and gasification to provide onsite destruction of PFAS and other emerging pollutants in sludge or biosolids warrants industry interest. The significant reduction or complete removal of these chemicals from sludge‐ or biosolids‐derived char may assist WRRF land application or beneficial reuse programs. While some work has been advanced to fully elucidate these chemicals' fate and transformation products in the residuals, oils, and gases from thermal treatment technologies to enable proper management, additional research is needed.

### PFAS

Significant emphasis has been placed on PFAS in sludge and biosolids due to their ubiquitous detection throughout the environment and recalcitrant nature (Winchell et al., 2022). Kim et al. (2015) conducted pyrolysis experiments with wastewater solids at laboratory scale at 300°C and 700°C, finding no significant change of PFAS in the biochar. By contrast, Kundu et al. (2021) demonstrated removal of all measured PFAS species in a municipal biosolid sample to non‐detect levels in char at temperatures ranging from 500°C to 600°C. Xiao et al. (2020) investigated the thermal stability of several PFAS on granular activated carbon (GAC) in various reducing atmospheres. The study observed that more than 80% of perfluorooctanoic acid (PFOA) and perfluorooctanesulfonic acid (PFOS) adsorbed on GAC was converted to fluoride ions at temperatures exceeding 700°C, and concentrations of both compounds were reduced by more than 99.9%. In a recent study, at temperatures of 500°C, Williams et al. (2021) found, in unreviewed research, three of 28 targeted PFAS species, of nearly 8,000 compounds in the PFAS family (USEPA, 2020), could be detected, all at less than 0.5 parts per billion (dry weight), or μg/kg, in the resulting biochar and the 28 PFAS were removed to non‐detect levels at 700°C. While this study showed promise for a handful of the PFAS that are potentially present in sludge or biosolids, further study is needed to validate PFAS removal at full‐scale as it is expected that the feedstock size, char hold time at pyrolysis temperature, and char porosity may impact heat diffusion through the feedstock and overall PFAS removal rates. Thoma et al. (2021) presented the first PFAS removal results from a full‐scale pyrolysis system processing dried biosolids. This research analyzed 41 PFAS in the dried biosolids and biochar. The study measured 21 PFAS ranging in concentration from 2 to 85 μg/kg in the dried biosolids. No PFAS were detected in the biochar, resulting in 81.3% to 99.9% removal when using the method detection limit concentration. The researchers identified hydrogen fluoride (HF), tetrafluoromethane (CF_4_), and hexafluoroethane (C_2_F_6_) in the flue‐gas after the thermal oxidizer but disagreement in results of different test methods and potential contamination prevented the authors from making definitive conclusions. Indirect measurements of the gas‐phase PFAS in the drain from the wet scrubber used for air emissions control erratically detected PFOA but all samples contained measurable amounts of perfluorooctane sulfonamide (PFOSA) with the latter not co‐identified in the dried biosolids. The authors hypothesized the PFOSA detection may have resulted from the more sensitive analytical method applied to the scrubber water compared to the dried biosolids but needs further investigation. In addition to the transformation through pyrolysis or gasification processes, the boiling point data for PFAS suggest they are retained with the solids through the prerequisite drying step; however, some transformation between compounds has been hypothesized by comparing the feedstock and dried product (Kim Lazcano et al., 2020).

Williams et al. (2021) analyzed the pyrolysis off‐gas stream for 31 specific PFAS in a bench scale setting. They found that the limited detection resulted in a combined mass removal efficiency of 84.4% and 95.6% of measured PFAS, including that found in the char at the two experimental temperatures. Pyrolysis and gasification provide opportunities to thermally decompose PFAS, which has been demonstrated in oxygen‐deficient conditions at temperatures as low as 600°C (Taylor & Yamada, 2003; Yamada et al., 2005). And, while the formation of elemental hydrogen and resulting thermal cracking reactions in a gasifier may enhance the destructive potential via hydrodefluorination, the presence of several organofluorine transformation products, including simple perfluorinated compounds, have also been identified in the off‐gas (Yamada et al., 2005). Further analysis of the extent to which transformation products are present is required to assess the efficacy of pyrolysis or gasification as a standalone treatment option. Further, many of the smaller, perfluorinated by‐products require higher temperatures for degradation than the parent compounds (Winchell, Ross, et al., 2021). Thus, generating substantially more mobile PFAS transformation products in the off‐gas is a risk.

The direct combustion of pyrolysis and gasification off‐gases provides a promising opportunity to fully oxidize PFAS transformation products with subsequent removal of the hydrogen fluoride in downstream air pollution controls. The direct combustion systems used with pyrolysis and gasification to date include thermal oxidizers such as those permitted as the best available control technology (BACT) for PFAS treatment from emissions at the Saint‐Gobain Performance Plastics (SGPP) and Chemours industrial facilities (Beahm, 2019; Focus Environmental Inc., 2020). These facilities are required to maintain a minimum combustion temperature of 980°C with initial residence times proposed from 0.75 to 1.2 s. Initial stack testing at Chemours suggests these operating conditions meet the 99.99% destruction efficiency of specific PFAS as mandated by permit (Focus Environmental Inc., 2020). In addition, targeted and non‐targeted PFAS analyses of pyrolyzer/gasifier off‐gas and thermal oxidizer flue gas utilizing fluorine balance techniques such as total organic fluorine (TOF) can be conducted to verify destruction (Winchell, Wells, et al., 2021). Therefore, developing and validating laboratory‐scale pyrolysis or gasification and a direct combustion system with commercial‐scale applications could provide the industry a valuable tool to evaluate various operating parameters at a smaller scale and reduced cost.

### Other chemicals

Unregulated chemicals, including PPCPs, steroids, hormones, and other emerging pollutants, have been detected in wastewater effluent, sludge, and biosolids (McClellan & Halden, 2010; Patel et al., 2019; USEPA, 2009). Some of these chemicals can be fully or partially degraded by conventional wastewater treatment processes, but others remain in the effluent or partition to sludge and biosolids (Kinney et al., 2006; Luo et al., 2014; Spongberg & Witter, 2008; Walters et al., 2010). Sorption potential was identified as a primary factor governing chemical persistence in biosolids (Heidler & Halden, 2008). PPCPs with log K_ow_ values greater than 5.2 or log K_oc_ values greater than 4.4 were predicted to have the greatest persistence in biosolids.

PPCPs frequently detected in biosolids include triclocarban, triclosan, miconazole, tetracycline, 4‐epitetracycline, norfloxacin, ciprofloxacin, doxycycline, paroxetine, and ofloxacin (Guerra et al., 2014; USEPA, 2009). McClellan and Halden (2010) determined mean concentrations of 72 PPCPs from 110 biosolids samples and identified triclocarban, triclosan, ciprofloxacin, ofloxacin, 4‐epitetracycline, tetracycline, minocycline, and diphenhydramine as having the highest concentrations. In 2016, however, the U.S. Food and Drug Administration issued a final rule establishing that 19 specific ingredients, including triclosan and triclocarban, were no longer generally recognized as safe and effective, prohibiting companies from marketing soaps as antibacterial if they contain one or more of these ingredients. Brose et al. (2019) reported that companies promptly removed these compounds from products with a 70% decrease in triclosan and triclocarban in per capita influent loading into seven WRRFs from 2012 to 2017. There was a corresponding 70% decrease in triclosan and an 80% decrease in triclocarban concentrations in biosolids.

Most studies evaluating the ability of pyrolysis to remove unregulated chemicals have been conducted using temperatures below 600°C. For example, Mercl et al. (2021) tested 69 pharmaceuticals from 27 drug classes in biosolids. They found that pyrolysis at 420°C resulted in concentrations for all pharmaceuticals below detection limits in the char. Similarly, Moško et al. (2021) reported that temperatures as low as 400°C were sufficient to transform amitriptyline, caffeine, carbamazepine, diclofenac, dosulepin, hydrochlorothiazide, ibuprofen, metoprolol, and saccharin to below detection limits. Ross et al. (2016) reported that pyrolysis removed the antimicrobials triclosan and triclocarban to below the quantification limits at 300°C and 200°C, respectively. They also found that nonylphenol, an endocrine‐disrupting compound, was removed at 600°C. Endocrine disruptors and hormones were effectively removed from biosolids with pyrolysis at temperatures as low as 400°C, with removal efficiency increasing with temperature (Hoffman et al., 2016; Moško et al., 2021). Ni et al. (2020) tested pyrolysis at temperatures between 150°C and 500°C and recommended at least 450°C to remove microplastics present in biosolids. PAHs and polychlorinated biphenyls (PCBs) were removed with pyrolysis; however, temperatures over 600°C were required to achieve removal efficiencies greater than 99.8% (Moško et al., 2021). Kimbell et al. (2018) demonstrated that pyrolysis of biosolids at 500°C and higher resulted in approximately 6‐log removal of antibiotic‐resistant genes. These studies suggest that pyrolysis is a practical treatment pathway for transforming unregulated contaminants. As discussed previously, the emerging pollutants of interest in the sludge or biosolids will likely be retained through the upfront drying step due to evaporative cooling; the process temperature is often maintained below 80°C.

The fate of emerging pollutants during the thermal treatment of sludge and biosolids is not fully understood. Although studies have shown that thermal treatment effectively transforms many unregulated chemicals from the solid phase, there is a lack of data regarding concentrations of these chemicals and their transformation products in the resulting oils and off‐gases. Studies have suggested that PPCPs, PAHs, PCBs, endocrine disruptors, and hormones either volatilize or decompose due to their physicochemical properties (Moško et al., 2021; Ross et al., 2016). Ross et al. (2016) found that compounds with higher vapor pressures were more likely to volatilize while compounds with lower vapor pressures had longer retention times and were more likely to be transformed; reductive dehalogenation was suggested as a mechanism for the transformation of triclocarban. Hu et al. (2020) investigated the concentration of 16 PAHs in pyrolysis products from different sewage sludges. They found that most PAHs were formed during pyrolysis and mainly ended up in the oil and off‐gas with higher temperatures, promoting more significant PAH formation in the oil. Like the fate of PFAS through pyrolysis, the coupling of a thermal oxidizer leads to extensive destruction of the volatilized PAH compounds, but further research must be completed to verify this finding.

## CONCLUSIONS

The suppliers surveyed as a part of this work prove that the interest in pyrolysis and gasification technologies to process WRRF sludge or biosolids continues despite historical challenges. The quintessential benefit of these technologies is reducing the amount of mass requiring subsequent management or disposal. This mass also has properties supporting beneficial reuse applications if reliable markets can be adequately developed. Potential transformation or destruction of emerging pollutants such as PFAS also increases their attractiveness to WRRF facilities looking for proactive solutions or hedging against future regulations. The processes identified can utilize the energy present in the sludge and biosolids to satisfy the thermal requirements.

While promising, these technologies are just entering the US market. Of the suppliers surveyed, three have a single system that can be considered full scale, while the others are in construction or pilot scale development. These suppliers have also noted several additional full‐scale facilities in construction or development, indicating the industry will soon have several examples to evaluate. Detailed evaluation of these facilities for several years after startup is recommended to determine whether operation and maintenance requirements, reliability, performance, energy recovery, and other aspects generally warrant widespread adoption of the technology. Nevertheless, with the current pyrolysis and gasification installations and those being planned, the wastewater treatment industry has three high‐temperature alternatives for sludge or biosolids processing.

## CONFLICT OF INTEREST

The authors declare no conflict of interest.

## AUTHOR CONTRIBUTIONS

**Lloyd Winchell:** Conceptualization; data curation; formal analysis; investigation; methodology; project administration; supervision. **John Ross:** Conceptualization; data curation; formal analysis; investigation; methodology; visualization. **Dominic Brose:** Conceptualization; data curation; formal analysis; investigation; methodology. **Thaís Pluth:** Conceptualization; data curation; formal analysis; investigation; methodology. **Katherine Bell:** Conceptualization; project administration.

## Data Availability

The data that support the findings of this study are available from the corresponding author upon reasonable request.
